# Impact of Smoking and Thiocyanate on Perchlorate and Thyroid Hormone Associations in the 2001–2002 National Health and Nutrition Examination Survey

**DOI:** 10.1289/ehp.10300

**Published:** 2007-07-09

**Authors:** Craig Steinmaus, Mark D. Miller, Robert Howd

**Affiliations:** Office of Environmental Health Hazard Assessment, California Environmental Protection Agency, Oakland, California, USA

**Keywords:** cotinine, interactions, iodine, perchlorate, thiocyanate, thyroid, tobacco smoke

## Abstract

**Background:**

Findings from a recent large study suggest that perchlorate at commonly occurring exposure concentrations may decrease thyroid hormone levels in some women. Decreases in thyroid hormone seen with perchlorate exposure could be even greater in people with concomitant exposure to agents such as thiocyanate that may affect the thyroid by mechanisms similar to those of perchlorate.

**Objectives and methods:**

We used data from the National Health and Nutrition Examination Survey to assess the impact of smoking and thiocyanate on the relationship between urinary per-chlorate and serum thyroxine (T_4_) and thyroid-stimulating hormone (TSH).

**Results:**

In women with urinary iodine levels < 100 μg/L, the association between the logarithm of perchlorate and decreased T_4_ was greater in smokers [regression coefficient (β) = −1.66, *p* = 0.0005] than in nonsmokers (β = −0.54, *p* = 0.04). In subjects with high, medium, and low cotinine levels, these regression coefficients were −1.47 (*p* = 0.0002), −0.57 (*p* = 0.03), and −0.16 (*p* = 0.59). For high, medium, and low thiocyanate tertiles they were −1.67 (*p* = 0.0009), −0.68 (*p* = 0.09), and −0.49 (*p* = 0.11). Clear interactions between perchlorate and smoking were not seen with TSH or with T_4_ in women with urinary iodine levels ≥ 100 μg/L or in men.

**Conclusions:**

These results suggest that thiocyanate in tobacco smoke and perchlorate interact in affecting thyroid function, and this effect can take place at commonly occurring perchlorate exposures. Agents other than tobacco smoke might cause similar interactions, and further research on these agents could help identify people who are particularly susceptible to perchlorate.

Perchlorate has been used by the aerospace industry as an oxidizer in solid rocket propellant, slurry explosives, road flares, and air bag inflation systems. Human environmental exposure can occur through food or water after industrial contamination or from perchlorate that is naturally occurring. In a recent analysis of data from the 2001–2002 National Health and Nutrition Examination Survey (NHANES), detectable levels of perchlorate were found in the urine of all 2,820 U.S. residents assessed (Blount et al. 2006b). Further analysis of the 2001–2002 NHANES data also identified an association between urinary levels of perchlorate and decreased levels of serum thyroxine (T_4_) in women with urinary iodine concentrations < 100 μg/L (Blount et al. 2006a). A remarkable feature of this finding is that it was identified at perchlorate concentrations much lower than those linked to thyroid effects in any previous study. In fact, NHANES was designed to provide a nationally representative sample. Thus, the perchlorate exposure levels of the subjects in this study are likely similar to exposures occurring in a large portion of the U.S. population. Another notable feature was the relatively large fraction of women who had urinary iodine levels in the range where these effects were found. That is, 35% of all women ≥ 12 years of age in NHANES 2001–2002 had urinary iodine levels < 100 μg/L.

High doses of perchlorate have been shown to competitively inhibit iodide uptake in the thyroid gland, and perchlorate has been used therapeutically for this effect (Stanbury and Wyngaarden 1952; Wyngaarden et al. 1952). Other agents, including nitrate in food and water and thiocyanate in food or from tobacco smoke, also affect the thyroid by the same mechanism (Braverman et al. 2005; Tonacchera et al. 2004; Wyngaarden et al. 1953). Thiocyanate is a metabolite of cyanide found in tobacco smoke, and increased serum thiocyanate levels are associated with increasing levels of smoking (Foss and Lund-Larsen 1986). Thiocyanate is also found in some foods including cabbage, broccoli, cassava, and rapeseed oil (Foss and Lund-Larsen 1986).

In this investigation, we used data from the 2001–2002 NHANES to assess whether thiocyanate or other substances in tobacco smoke may interact with iodine and low levels of perchlorate in affecting T_4_ and thyroid-stimulating hormone (TSH) (National Center for Health Statistics 2002). The purpose of this investigation was to help increase our understanding of the mechanisms of perchlorate toxicity, to evaluate whether associations between perchlorate and thyroid hormones may differ between smokers and nonsmokers (or categories of cotinine and thiocyanate), and to help identify subpopulations that may be more susceptible than others to the thyroid effects of perchlorate. These issues could have tremendous public health significance given the large numbers of people who appear to be exposed to perchlorate and have low iodine intake. The recent finding linking low levels of urinary perchlorate to reduced thyroid function, and the possibility that even minor decrements in thyroid function could affect early-life cognitive development, highlight the potentially serious impact of perchlorate exposure.

## Materials and Methods

NHANES is a national survey of health, nutrition, and sociodemographic information involving a complex multistage probability sampling design conducted by the Centers for Disease Control and Prevention’s National Center for Health Statistics (2002). In NHANES 2001–2002, levels of perchlorate, cotinine, and thiocyanate were analyzed in a representative one-third subsample of all subjects. For all subjects ≥ 12 years of age, these data were combined with data on levels of serum T_4_, serum TSH, smoking status, and other variables that might affect perchlorate–thyroid associations. These included race (Mexican American, other Hispanic, non-Hispanic white, non-Hispanic black, or other, each entered as a dichotomous variable), age (years), iodine status, serum albumin (grams per deciliter), body mass index (BMI) (kilograms per square meter), 24-hr caloric intake (kilocalories per day from 24-hr dietary recall data), pregnancy status (based on urinary pregnancy test results and self-reports), postmenopausal status (based on self-report, time since last period, and presence or absence of other reasons for missed menstruation), premenarche status (self-report), serum c-reactive protein (milligrams per deciliter), hours of fasting before serum collection (self-report), urinary nitrate (micrograms per liter), current lactation (dichotomous based on self-report), and use of medications known to affect the thyroid (e.g., levothyroxine, methimazole, propylthiouracil, beta blockers, and estrogens) (dichotomous based on self-reported use). A description of the possible impacts these variables may have on the relationship between perchlorate and thyroid function has been provided by Blount et al. (2006a).

In NHANES 2001–2002, information on self-reported smoking status was provided in two separate sections, the questionnaire and the medical examination component (MEC). For the study presented here, subjects were divided into four groups: current smokers, recent former smokers, long-term former smokers, and never-smokers. Current smokers were defined as those who reported that they smoked cigarettes at the time of the survey (questionnaire) or reported that they had smoked at least 20 cigarettes in the preceding 30 days (MEC). Never-smokers were defined as those who reported that they had not smoked at least 100 cigarettes in their life (questionnaire) or that they had never tried cigarette smoking (MEC). Recent former smokers were defined as those who quit smoking within 12 months of the survey. Long-term former smokers were defined as those who quit > 12 months before the survey. Because no difference between long-term former smokers and never-smokers was found in the association between T_4_ or TSH and perchlorate, these two groups were combined into one group labeled “nonsmokers” for this study. Because the latency of the impact of tobacco smoke on thyroid function is unknown, all recent former smokers were excluded from analyses comparing smokers and nonsmokers. Approximately 10% of the subjects did not provide adequate information to assess smoking status. Few women were regular users of cigars, chewing tobacco, or snuff, and excluding regular users of these products had no impact on the analyses.

The methods used to collect and perform laboratory analyses of perchlorate, iodine, T_4_, TSH, cotinine, and thiocyanate, and the quality control procedures involved in NHANES 2001–2002, are described in detail elsewhere (National Center for Health Statistics 2002). Briefly, serum samples collected in 2001 were assayed for TSH using a microparticle immunoassay and for T_4_ using a Hitachi 704 chemistry analyzer (Hitachi Chemical Diagnostics, Mountain View, CA). Samples collected in 2002 were assayed for T_4_ and TSH using a chemiluminescent immunoassay (Access Immunoassay Systems; Beckman Instruments, Fullerton, CA). Urine samples were analyzed for perchlorate, thiocyanate, and nitrate using ion chromatography tandem mass spectrometry (Blount 2006b). Iodine was analyzed using inductively coupled plasma mass spectrometry (National Center for Health Statistics 2002).

Using data from NHANES 2001–2002, Blount et al. (2006a) identified an association between the logarithm of perchlorate concentration in urine (log perchlorate) and decreased serum T_4_ in women with urinary iodine < 100 μg/L (β = −0.89, *p* < 0.0001), but not in women with higher urinary iodine levels (β = 0.22, *p* = 0.55). Associations between log perchlorate and increases in the logarithm of serum TSH (log TSH) were also identified, both in women with urinary iodine levels above and below 100 μg/L (β = 0.11, *p* = 0.02; and β = 0.12, *p* = 0.001, respectively). The urinary iodine stratification level of 100 μg/L was selected by Blount et al. because the World Health Organization (WHO) has defined populations with median urinary iodine excretion > 100 μg/L as having sufficient intake (WHO 1994). This stratification level was chosen for the analyses presented here for the same reason and so that our results could be compared more directly with those of Blount et al. (2006a)

We assessed serum cotinine in this study because it is a metabolite of nicotine and may be a more accurate indicator of current smoking status than self-reported smoking (Wells et al. 1998). We categorized cotinine concentrations into three levels for this study. Serum cotinine levels > 10 ng/mL were classified as high, whereas levels below the detection level of 0.015 ng/mL were classified as low. All levels between these cutoff points were classified as medium. Using a cutoff of 15 ng/mL to define the high cotinine category had little impact on our results. Thiocyanate categories were based on the sex-specific tertiles.

All statistical analyses were performed with SAS version 9.1 (SAS Institute Inc., Cary, NC) using SAS procedures surveyreg, %sregsub, and surveymeans. The following model was used: T_4_ or log TSH = intercept + β _1_(log perchlorate) + β _2_(log creatinine) + β _3_(iodine category) + β _4_(iodine category × log perchlorate) +β_5_(smoking category) + β_6_(smoking category × log perchlorate) + β *_n_*(*n* = other potential confounding variables). In this model iodine category is defined as < 100 μg/L or not, and smoking category is defined either as current versus nonsmokers or as classes of cotinine or thiocyanate as defined above. The model allows estimates of the effect of smoking category and the interaction of perchlorate with smoking category, after adjustment for creatinine and iodine category. Similarly, estimates of the effect of iodine category and the interaction of iodine category with perchlorate are made after adjustment for creatinine and smoking category. Initially, we performed univariate analyses to assess the relationship between urinary perchlorate, all potential covariates, and serum T_4_ and log TSH. We also performed univariate analyses and data plots to identify outliers and to assess distributions. Variables such as urinary perchlorate, urinary creatinine, and serum TSH were log 10-transformed to normalize distributions. In analyses involving surveymeans, urinary perchlorate residuals adjusted for creatinine were calculated using the method described by Willet and Stampfer (1998).

Initial univariate and multivariate analyses showed no clear evidence of interaction among smoking, cotinine, thiocyanate, and urinary perchlorate on serum T_4_ or log TSH levels in men or in women with urinary iodine levels > 100 μg/L, so we focus on women with urinary iodine < 100 μg/L. Women who reported a history of thyroid disease or use of thyroid medications were excluded. Two women with very high serum TSH levels (43 and 68 IU/L) and one woman with a very high serum T_4_ level (27 μg/dL) were also excluded.

We constructed separate regression models for serum T_4_ and log TSH as the dependent variables. Model building followed the general principles presented by Hosmer and Lemeshow (2000). Independent variables that were associated with T_4_ or log TSH with *p* < 0.20 were added to and retained in the model that included the log perchlorate and log creatinine. Excluded variables were added back to the model one at a time to assure that they did not have important impacts on the perchlorate regression coefficient.

## Results

Of the 11,039 subjects in NHANES 2001–2002, 5,708 were women and 4,137 of these were ≥ 12 years of age. Of these, 1,203 (29%) had data on serum T_4_, serum TSH, and urinary perchlorate, iodine, and creatinine. Three of these had extreme values of T_4_ or TSH, and 91 reported a history of thyroid disease or use of thyroid medication. Of the remaining women, 385 (35%) had urinary iodine levels < 100 μg/L. Table 1 provides mean levels of various parameters for smokers and nonsmokers.

Table 2 shows the regression coefficients for the association between serum T_4_ and log perchlorate in women with urinary iodine levels < 100 μg/L. The adjusted regression coefficient in all women with low urinary iodine was −0.73 (*p* = 0.004). The adjusted regression coefficients between T_4_ and log perchlorate in current smokers and nonsmokers were −1.66 (*p* = 0.0005) and −0.54 (*p* = 0.04), respectively. The regression coefficient for the product term including log perchlorate and smoking category was 1.12 (*p* = 0.008), consistent with a biologic interaction. Regression coefficients between T_4_ and log perchlorate were higher in women with serum cotinine levels > 10 ng/mL than in women with cotinine levels below the detection limit, and higher in women with thiocyanate levels in the upper tertile than in women with levels in the lower tertile. We observed only small differences between the adjusted and unadjusted regression coefficients in most analyses. The differences in T_4_–log perchlorate regression coefficients seen between smoking categories changed only slightly when different iodine cutoff points were used. For example, using a cut-off point of 75 μg/L and 125 μg/L, the T_4_–log perchlorate regression coefficients for smokers and nonsmokers were −1.58 and −0.48, and −1.42 and −0.33, respectively. No clear associations between urinary perchlorate and T_4_ or interactions with smoking, cotinine, or thiocyanate were found in men, or in women with urinary iodine levels ≥ 100 μg/L (data not shown).

Table 2 shows the regression coefficients for the association between log TSH and log perchlorate in women with urinary iodine levels < 100 μg/L. In all women in this group, the regression coefficient was 0.13 (*p* = 0.02). The corresponding coefficients in current smokers and nonsmokers were 0.13 (*p* = 0.23) and 0.11 (*p* = 0.009). Consistent differences in the regression coefficients between urinary perchlorate and serum TSH were not seen across subgroups of serum cotinine or thiocyanate.

The associations between T_4_ and smoking, thiocyanate, and cotinine, independent of urinary perchlorate, were assessed in women with urinary iodine < 100 μg/L. In univariate analyses, no associations were found between the logarithm of serum cotinine and serum T_4_ or log TSH (for T_4_: *n* = 382, β = 0.03, *p* = 0.73; for log TSH: *n* = 382, β = −0.02, *p* = 0.26). In analyses adjusted for creatinine, no statistically significant associations were found between log thiocyanate and T_4_ or log TSH (for T_4_: *n* = 384, β = −0.17, *p* = 0.46; for TSH: *n* = 384, β= −0.05, *p* = 0.21).

Table 3 shows mean serum T_4_ and log TSH levels in women with and without three potential risk factors for reduced uptake of iodine into the thyroid gland and thus reduced production of thyroid hormone. These are: *a*) urinary iodine < 100 μg/L (the median urinary iodine concentration in a population at which no endemic goiter is found) (Vanderpas 2006; WHO 2004)), *b*) current smoking, and *c*) urinary perchlorate (creatinine adjusted) above the median. Mean T_4_ levels were lower in women who had all three risk factors than in those with none (7.16 vs. 8.41 μg/dL, *p* = 0.04). Mean log TSH was higher in the group with these risk factors than in those without [0.24 vs. 0.11 (IU/L), *p* = 0.001]. These differences were smaller and not statistically significant when the perchlorate risk factor was removed. That is, the mean T_4_ level in women smokers with iodine < 100 μg/L (regardless of urinary perchlorate level) was 8.58 μg/dL [95% confidence interval (CI), 8.11 to 9.05, *n* = 66] whereas the mean T_4_ in nonsmokers with iodine ≥ 100 μg/L (regardless of urinary perchlorate level) was 8.39 μg/dL (*n* = 406, 95% CI, 8.06 to 8.72; *p* = 0.63). The mean log TSH values in these two groups were 0.12 μg/dL (95% CI, −0.00 to 0.24) and 0.16 μg/dL (95% CI, 0.13 to 0.19; *p* = 0.36).

## Discussion

A previous analysis of NHANES 2001–2002 showed that commonly found urinary perchlorate levels may be associated with decreases in serum T_4_ and increases in TSH in some women (Blount et al. 2006a). Our independent analysis of the same raw data confirms these findings and has also found that in women with low urinary iodine, the association between urinary perchlorate and decreased serum T_4_ is stronger in smokers than in nonsmokers and stronger in those with high urinary thiocyanate levels than in those with low thiocyanate levels. These findings provide evidence that perchlorate at relatively low levels can interact with tobacco smoke or thiocyanate to diminish iodine uptake and decrease thyroid function. The large magnitude of the differences identified suggest that these findings are not owing to chance, and the finding of similar effects across different measures of exposure (i.e., self-reported smoking, serum cotinine, and urinary thiocyanate) provides further evidence that these results represent true effects.

The findings identified in this study are biologically plausible. That is, they are consistent with the mechanisms by which perchlorate, iodine, and thiocyanate are known to affect thyroid function. Perchlorate competitively inhibits the sodium iodide symporter (NIS), the membrane protein that actively transports iodide into the thyroid follicular cell for thyroid hormone production (Stanbury and Wyngaarden 1952; Wyngaarden et al. 1952). Sufficient suppression of iodide uptake can diminish production of thyroid hormone. Smoking has been linked to increases in various thyroid diseases (Vestergaard 2002), although data on the relationship between smoking and serum levels of thyroid hormones have been mixed (Bertelsen and Hegedus 1994). Smoking does increase serum levels of thiocyanate (Foss and Lund-Larsen 1986), which, like perchlorate, also competitively inhibits the NIS (Tonacchera et al. 2004). Thiocyanate has also been shown to increase the risk for development of goiter, both in those with moderate to severe iodine deficiency (< 50 μg/g creatinine) and in those within the lower range of adequate iodine intake (100 μg/g creatinine) (Brauer et al. 2006). Based on their common effects, it is not surprising that thiocyanate from smoking could interact with perchlorate to inhibit the NIS and subsequently decrease thyroid hormone production and that this effect would be greater in those with lower iodine levels.

A remarkable aspect of these findings is that the perchlorate levels in NHANES 2001–2002 are generally much lower than levels shown not to affect thyroid function in previous studies. In the 385 women with low iodine in our study, 50% had urine perchlorate levels < 1.7 μg/L and 90% had levels < 5.7 μg/L. Several experimental and observational studies have reported that perchlorate had no effect on T_4_ or TSH levels at exposure levels orders of magnitude higher than the median levels found in our study (Braverman et al. 2005, 2006; Greer et al. 2002; Lamm and Doemland 1999; Lamm et al. 1999a, 1999b, 2002; Tellez et al. 2005). There are several possible explanations for this discrepancy. One is that most previous studies did not evaluate effects in women with low iodine. It is possible that iodine levels must be sufficiently low for environmental levels of perchlorate and thiocyanate to overcome compensatory mechanisms that maintain thyroid hormone. This is consistent with a previous study showing that goiters due to the consumption of thiocyanate in cassava were more likely when iodine levels were low and were reversed with iodine supplementation (Delanghe et al. 1982). It is also possible that the effects on T_4_ we identified in women with low urinary iodine also occur in women with higher iodine levels but are less strong and the statistical power to identify them has been too low in most studies. Other possible reasons are that occupational studies have involved primarily men and, as was found in our study, effects may predominate in women; that experimental studies may have missed effects because dosing durations were too short; and that ecologic studies may have missed effects because of the greater misclassification of perchlorate exposure that can occur when exposure is assessed ecologically rather than individually. We do not know why the effects we identified were seen only in women. Women do have greater incidences of some iodine-related and other thyroid conditions (Laurberg et al. 2000), and estradiol has been shown to inhibit TSH-stimulated expression of the sodium–iodide symporter in rats (Furlanetto et al. 1999). It is possible that these effects may be related to our finding effects in women but not in men.

The results of this study are based on a single assessment of urinary perchlorate, urinary iodine, and serum thyroid hormones. Previous studies have shown that after short-term perchlorate administration in humans, most perchlorate is excreted in the urine within 48–72 hr (Greer et al. 2002; Selivanova and Arefaeva 1986). Other studies have shown that thyroid hormone levels, especially TSH, and iodine levels can have marked intraindividual variability over time (Hollowell et al. 1998; Surks et al. 2005). Because of this variability and the relatively short half-life of perchlorate, single measurements of these analytes might not reflect true long-term levels. Another issue that could lead to misclassification is that serum T_4_ was measured as total T_4_ rather than as free T_4_ (the physiologically available form). Importantly, though, biologic samples in NHANES 2001–2002 were collected and analyzed similarly in all subjects, independent of perchlorate, thyroid, iodine, or smoking status. Thus, any misclassification of these exposure or outcome variables is likely to be nondifferential, and therefore likely to bias estimates of association toward the null, not toward the positive effects identified in this study (Rothman and Greenland 1998).

The presence of certain factors such as estrogen use, the presence of thyroid disease, exercise, menstruation disturbances, or antithyroid antibodies could potentially confound the association between perchlorate and thyroid hormone levels. However, confounding variables must be related to both the exposure and the outcome. Because many of the variables linked to serum thyroid hormone levels are not necessarily associated with urinary perchlorate concentrations, they are not likely to be confounding variables in our analyses. Some variables could be associated with both thyroid hormone levels and urinary perchlorate and therefore could have acted as confounders. However, many potential confounders were controlled for either by adjusting for them in the analyses (e.g., estrogen use) or by applying restriction criteria (e.g., excluding subjects with known thyroid disease).

The presence of antithyroid antibodies is a risk factor for hypothyroidism and decreased T_4_, but were not measured in NHANES 2001–2002 and therefore not included in our analyses. The actual impact of this is unknown. However, an analysis of a previous NHANES data set (NHANES III) found that antithyroid antibodies were less prevalent in smokers (11%) than in nonsmokers (18%) (Belin et al. 2004). This suggests that, based on antithyroid antibody status alone, smokers would tend to have higher T_4_ levels than nonsmokers. In the study reported here, we found somewhat of the opposite effect. That is, we found that per-chlorate was associated with decreased T_4_, and the strength of this decrease was greater in smokers than in nonsmokers. This suggests that if we were able to account for antithyroid antibodies in our analyses, this could actually strengthen, not weaken, the associations we identified for smoking.

The results of some of our analyses are slightly different than those reported by Blount et al. (2006a), although the results of these two studies are close and the differences have no impact on the conclusions drawn from either study. Our analysis was done independently from Blount et al., so these differences are likely related to differences in the definitions and categorization of some variables (e.g., postmenopause or premenarche status), the use of different statistical programs, and different methods of selecting independent variables to remain in the final regression models. Despite these differences, the results of these two studies are similar. For example, for women with low urinary iodine the adjusted regression coefficient between log perchlorate and serum T_4_ was −0.89 (SE = 0.18, *p* < 0.0001, *n* = 348) in Blount et al. (2006a) and −0.73 (SE = 0.22, *p* = 0.004, *n* = 362) in this study. The similarity of these results, despite different statistical methods and independent analyses, supports the validity of the methods used in both studies.

Tobacco smoke, with its > 4,000 chemicals, is associated with many abnormalities of thyroid function likely occurring via multiple mechanisms (Utiger 1995). Although we found evidence of interaction between smoking and perchlorate on reducing serum T_4_ levels, no clear interaction was seen on TSH. As seen in Table 2, the magnitudes of the regression coefficients between log perchlorate and log TSH were similar in nonsmokers and smokers, suggesting that the impacts of perchlorate on TSH are similar regardless of smoking status. In many conditions, suppression of T_4_ is accompanied by a compensatory rise in serum TSH. However, this is not always the case. For example, iodine deficiency is characterized by decreases in serum T_4_ with normal or slightly elevated levels of serum triiodothyronine (T_3_) and normal levels of serum TSH (Obregon et al. 2005). It has been shown that with iodine deficiency T_4_ is deiodinated to T_3_ (LaFranchi 2004). Based on this, it has been suggested that although iodine deficiency may cause a decrease in serum T_4_ levels, an associated increase in T_3_ exerts a negative feedback limiting any compensatory rise in TSH (Maruta and Greer 1988). This could explain why we found clear differences across smoking, cotinine, and thiocyanate categories for T_4_ but not for TSH.

There are other possible reasons why we found less effect on TSH. For example, a previous NHANES survey found that smokers had lower TSH levels than nonsmokers, possibly related to the greater prevalence of serum thyroid autoantibodies among non-smokers (Belin et al. 2004). Although the data from other studies regarding the impacts of smoking and thyroid function have been mixed (Hegedus et al. 1985; Muller et al. 1995; Petersen et al. 1991; Sepkovic et al. 1984), it is possible that smoking causes an overall suppression of TSH that counterbalances any TSH stimulation caused by reductions in serum T_4_. Another reason why effects on TSH may not be found is that intra- and interindividual variability is generally much greater for serum TSH than for serum T_4_, and this variability diminished the ability of this study to identify associations with a single assessment of TSH.

The public health significance of this study lies in *a*) the large number of people in the United States who appear to be exposed to perchlorate, *b*) the large number of women with low iodine levels, *c*) the variety of factors that can inhibit the sodium iodide symporter, and, *d*) the potential importance of relatively small changes in thyroid hormone levels. With regard to perchlorate exposure, NHANES was designed to provide information on a nationally representative level. Thus, the perchlorate exposure levels of the subjects in this study are supposed to represent those in the large majority of people in the United States. Although statistically significant findings in this study were seen only in women with urinary iodine levels < 100 μg/L, this group represents a large fraction of the U.S. and world population. In NHANES 2001–2002, 35% of all women ≥ 12 years of age had iodine levels < 100 μg/L.

Importantly, our findings may not be relevant just for smokers. We did find effects on TSH in both smokers and non-smokers. In addition, we found a borderline statistically significant effect on T_4_ in nonsmokers and a statistically significant effect in subjects with urinary cotinine levels associated with environmental tobacco smoke exposure (i.e., our medium cotinine category). Also, thiocyanate not only results from smoking but also is present in various foods including cruciferous vegetables and cassava. Elevated levels of thiocyanate have been noted in the milk of cows that were fed rapeseed (a member of the Cruciferae family), with resulting decreases in iodine in milk presumably from inhibition of the sodium–iodide symporter expressed in the lactating breast (Laurberg et al. 2002). Similarly, iodine levels in breast milk are decreased in human mothers who smoke (Laurberg et al. 2004). Other chemicals known to inhibit the thyroid sodium–iodide sym-porter include nitrates [which are estimated to contaminate 22% of domestic wells throughout the United States above the U.S. EPA maximum concentration limit (Ward et al. 2005)] and iodine in excess (Riesco-Eizaguirre and Santisteban 2006; Wolff and Chaikoff 1948). *In vitro* data have shown that perchlorate, nitrates, and thiocyanate may have additive effects on inhibiting the sodium–iodide symporter (Tonacchera et al. 2004). In preliminary analyses, we did not find evidence of strong associations between urinary nitrate levels and thyroid function or clear interactions with perchlorate. Whether this may be caused by the use of urinary nitrate rather than serum or dietary nitrate, differences between *in vivo* and *in vitro* effects, or some other reason is unknown and requires investigation beyond the scope of this paper. Regardless, the *in vitro* data on the interactions between these agents, combined with our findings regarding thiocyanate, provide some evidence that the effect of perchlorate on thyroid function can be increased in people exposed to similarly acting agents. A previous concern about the interpretation of the report by Blount et al. (2006a) was that they identified associations with perchlorate but not with other goitrogens (American Thyroid Association 2006). The study reported here adds importantly to the evaluation of the NHANES data by showing that other goitrogens, specifically thiocyanate and smoking, can also affect thyroid function.

The effects we identified, although relatively small, could have important implications in people who have borderline low T_4_ levels for reasons other than exposure to perchlorate. In these potentially susceptible sub-groups, additional reduction in T_4_ levels caused by perchlorate could increase their risks of developing signs or symptoms of hypothyroidism. Some research indicates that even small decrements in maternal free T_4_ (fT_4_) during the first trimester, including those within what is generally considered the “normal” range (lowest 10%), can be associated with impaired neuropsychologic development in the child (Haddow et al. 1999; Kooistra et al. 2006; Pop et al. 2003). This suggests that any exposure that would result in altered production or utilization of fT_4_ should be considered a potentially detrimental effect. Sizable subpopulations for which underlying fT_4_ levels may be low or borderline include women with iodine deficiency or thyroid peroxidase antibodies. These women constitute a large subpopulation potentially sensitive to the additional burdens of thiocyanate (from smoking or diet) and perchlorate.

## Conclusions

The results of this study suggest that thiocyanate and smoking can have marked impacts on the association between perchlorate, iodine uptake in the thyroid, and the production of T_4_. The results also provide an example of how an environmental chemical exposure could potentially interact with nutritional and lifestyle factors, including smoking, iodine sufficiency, and thiocyanate intake, to affect an important health outcome. In a time when chemical risk assessments are under mandate to identify and protect susceptible subpopulations, this study illustrates the need to evaluate interactions of not only chemicals but lifestyle choices, nutritional status, medications, and other factors. The results of this and other studies suggest that thyroidal effects of perchlorate are seen at commonly occurring perchlorate levels and fairly common iodine levels, and therefore could be affecting a large segment of the U.S. population. Further research is needed to confirm these findings and to identify other groups who may be particularly susceptible to the health effects of perchlorate, and information on these groups should be incorporated into public health policies aimed at preventing perchlorate toxicity.

## Figures and Tables

**Table 1:** Mean (± SE) levels of perchlorate, iodine, T_4_, logTSH, and other variables in smoking and nonsmoking women with urinary iodine < 100 μg/L, 2001–2002 NHANES.

	Current smokers	Nonsmokers
No.	66	260
Perchlorate (μg/L)	2.52 ± 0.55	3.15 ± 0.88
Iodine (μg/L)	51.7 ± 3.7	54.3 ± 2.2
Thiocyanate (μg/L)	4,344 ± 646	813 ± 64
Cotinine (ng/mL)	201.5 ± 15.7	1.1 ± 0.6
T_4_ (μg/dL)	8.6 ± 0.2	8.2 ± 0.2
logTSH (μg/dL)	0.12 ± 0.06	0.14 ± 0.03

**Table 2:** Association between the logarithm of urinary perchlorate (μg/L) and serum T_4_ (μg/dL) and the logarithm of TSH (μg/dL) in women with urinary iodine < 100 μg/L,^a^ 2001–2002 NHANES.

	T_4_^b^				Log TSH^c^			
	No.	β	SE	*p*-Value	No.	β	SE	*p*-Value
All	362	−0.73	0.22	0.004	369	0.13	0.05	0.02
Smoking^d^								
Current	63	−1.66	0.37	0.0005	62	0.13	0.11	0.23
Nonsmoker	245	−0.54	0.23	0.04	45	0.11	0.03	0.009
Cotinine (serum)								
High (> 10 ng/mL)	64	−1.47	0.30	0.0002	68	0.15	0.08	0.09
Medium^e^	185	−0.57	0.25	0.03	192	0.10	0.06	0.09
Low (ND)	101	−0.16	0.29	0.59	106	0.11	0.05	0.04
Thiocyanate (urine)^f^								
High (> 1,800 μg/L)	78	−1.67	0.40	0.0009	82	0.13	0.09	0.19
Medium	107	−0.68	0.37	0.09	108	0.20	0.04	0.0003
Low (< 751 μg/L)	176	−0.49	0.30	0.11	178	0.10	0.05	0.06

Abbreviations: ND, nondetectable; β , regression slope; *p*, two-sided *p*-value.

a This value was selected because a urinary iodine level > 100 μg/L is the WHO definition of sufficient iodine intake in populations (WHO 1994).

b T_4_ models were adjusted for fasting time, kilocalories, BMI, c-reactive protein, nitrate, race, estrogen use, and pregnancy. T_4_ model with cotinine was also adjusted for menopause status.

c LogTSH models were adjusted for age, fasting time, body mass index, race, premenarche, and lactation. LogTSH model with smoking status was also adjusted for menopause status.

d Smoking data were not available on all women, and recent former smokers are excluded.

e Medium category includes all subjects with serum cotinine levels between 10 ng/mL and nondetectable.

f Based on tertiles in all women ≥ 12 years of age.

**Table 3:** Comparing mean T_4_ (μg/dL) and the logarithm of TSH (IU/L) values in groups of women.

	T_4_		Log TSH	
	Mean	95% CI	Mean	95% CI
Group 1 (*n* = 21)	7.16	6.28–8.05	0.24	0.13–0.36
Group 2 (*n* = 161)	8.41	7.96–8.86	0.11	0.08–0.14
Difference	1.25		0.13	
*p*-Value	0.04		0.001	

Group 1: Current smokers, perchlorate residual (μg/L) > median, and urinary iodine < 100 μg/L. Group 2: Never-smokers, perchlorate residual (μg/L) ≤ median, and urinary iodine ≥ 100 μg/L. Urinary perchlorate residuals are adjusted for creatinine using the method described by Willet and Stampfer (1998).
